# Horizontal Transmission of *Metarhizium anisopliae *in Fruit Flies and Effect of Fungal Infection on Egg Laying and Fertility

**DOI:** 10.3390/insects4020206

**Published:** 2013-05-29

**Authors:** Susan Dimbi, Nguya K. Maniania, Sunday Ekesi

**Affiliations:** 1 Crop Protection Division, Tobacco Research Board, PO Box 1909, Harare, Zimbabwe; E-Mail: sdimbi@kutsaga.co.zw; 2 International Centre of Insect Physiology and Ecology (ICIPE), PO Box 30772-00100, Nairobi, Kenya; E-Mail: sekesi@icipe.org

**Keywords:** *Ceratitis capitata*, *C. fasciventris*, *C. cosyra*, fruit fly, *Metarhizium anisopliae*, horizontal transmission, fungal infection, egg laying, fertility

## Abstract

Fly-to-fly transmission of conidia of the entomopathogenic fungus *Metarhizium anisopliae *and the effect of fungal infection on the reproductive potential of females surviving infection were investigated in three fruit fly species, *Ceratitis cosyra*, *C. fasciventris*, and *C. capitata*. The number of conidia picked up by a single fruit fly was determined in *C. cosyra. *The initial uptake (Day 0) of conidia by a single fly was approx. 1.1 × 10^6^ conidia after exposure to the treated substrate. However, the number of conidia dropped from 7.2 × 10^5^ to 4.1 × 10^5^ conidia after 2 and 8 h post-exposure, respectively. The number of conidia picked up by a single fungus-treated fly (“donor”) varied between 3.8 × 10^5^ and 1.0 × 10^6^ in the three fruit fly species, resulting in 100% mortality 5–6 days post-exposure. When fungus-free flies of both sexes (“recipient” flies) were allowed to mate with “donor” flies, the number of conidia picked up by a single fly varied between 1.0 × 10^5^ and 2.5 × 10^5^, resulting in a mortality of 83–100% in *C. capitata*, 72–85% in *C. cosyra* and 71–93% in *C. fasciventris *10–15 days post-inoculation*. *There was an effect of fungal infection on female egg laying in the three species of fruit flies as control flies laid more eggs than fungus-treated females. The percentage reduction in fecundity in flies infected with *M. anisopliae* was 82, 73 and 37% in *C. capitata*, *C. fasciventris *and *C. cosyra*, respectively. The results are discussed with regard to application in autodissemination techniques.

## 1. Introduction

Several fruit fly species including *Ceratitis cosyra* (Walker), *C. fasciventris* (Bezzi), *C. rosa* (Karsch), *C. anonae*, *C. capitata* Weid. and *Bactrocera invadens *Drew Tsuruta and White (Diptera: Tephritidae), constitute a major constraint to mango production in Africa [1,2]. Losses of between 10–25% in the orchards of professional producers and between 30–80% in smallholder orchards have been reported [3]. The common control measures includes the use of chemical insecticides (such as Malathion 50% E.C. and Spinosad 25% E.C.) in bait spray for adult control and soil treatment with insecticide (such as Diazinon 50 E.C.) beneath host trees to kill fly larvae and puparia [4,5,6]. In many sub-Saharan Africa countries, there are no baits registered for fruit fly control and the majority of the farmers affected by fruit infestation practice broad-spectrum application of chemical insecticides, which often leads to fruit contamination and rejection by the export markets. The recent introduction of a uniform and strict quarantine and maximum residue level regulations (MRLs) by most fruit importing countries necessitates the development of alternatives to chemical control. Entomopathogenic fungi (EPF) are among the alternatives that are being developed at the International Centre of Insect Physiology and Ecology (*icipe*). A two-pronged approach is being considered [6]: The first targets pupariating larvae and puparia [3,7] and the second one targets adult flies [8]. In the first approach, a granular formulation of the fungus is inoculated under the mango canopy where it creates a hostile environment for pupariating larvae and puparia initiating high mortalities at the target stage [6,7,9]. In the second approach, dry conidia of the fungus are combined with food attractant in a baiting station or autoinoculation devices. Adult flies that are attracted to the device are contaminated with fungal conidia before they return to the environment where they can disseminate entomopathogens among wild populations [6,8]. Fundamental to the latter approach is the efficient horizontal transmission of the pathogen among individuals within fly populations. Within the same facet, the possibility of using sterile males to disseminate fungal conidia among fruit fly populations is being explored in Mexico in an area-wide-integrated pest management (IPM) that integrates sterile insect technique (SIT) [10]. Horizontal transmission of fungal conidia from treated to healthy individuals has been demonstrated in several dipteran flies [11,12,13,14,15,16,17]. In addition to mortality caused by EPF, sublethal effects of fungal infections can reduce the reproductive potential of insects including dipteran flies [17,18,19,20,21]. In microbial control of insect pests, secondary effects of the pathogen infection coupled with mortality may therefore play an important role in the management of the pest [22]. In the present study we investigate whether fungus-treated adult fruit flies can transfer conidia to fungus-free flies during mating and contact, and the effects of fungal infection on fecundity in females of three fruit fly species, *C. cosyra*, *C. fasciventris *and *C. capitata*.

## 2. Materials and Methods

### 2.1. Insects

Colonies of the three fruit fly species tested; *C. cosyra*, *C. capitata* and *C. fasciventris *were mass reared at the International Centre of Insect Physiology and Ecology (*icipe*). The initial colonies of *C*. *capitata *and *C*. *fasciventris* originated from coffee, *Coffea arabica* Linnaeus, collected from farms in the Central Highlands of Kenya at Ruiru (1°5.72' S; 36°54.22' E; 1,609 m above sea level) and Rurima (0°38.39' S; 37°29' E; 1,228 m above sea level). The *Ceratitis cosyra *colony was derived from collections obtained from mango, *Mangifera indica *Linnaeus and marula, *Sclerocarya birrea* (A. Rich.) Hochst. at Nguruman, Kenya (1°47' S; 36°05' E; 700 m above sea level). The larvae of the three species were reared on a carrot-sugar based artificial diet modified from Hooper [23] for 36–40 generations. Adult flies were maintained on a 4:1 volumetric mixture of sugar and enzymatic yeast hydrolysate [(Mumias Sugar Co., Kenya) and yeast hydrolysate (Yeast hydrolysate enzymatic, USB, Corporation, Cleveland, OH, USA)]. The adult flies were reared in ventilated Plexiglas cages (60 × 35 × 70 cm) at temperatures between 24–28 °C and under 12:12 L:D photoperiod. Reproductively active flies at 10–14 days of age were used in the study.

### 2.2. Fungal Isolate

*Metarhizium anisopliae* isolate ICIPE 62 used in this experiment was obtained from the *icipe’s* Arthropod Germplasm Centre. The fungus was maintained on Sabouraud dextrose agar (SDA) plates at room temperature (25 ± 4 °C). Conidia were harvested from the surface culture by scraping with a scalpel. Viability tests were carried out using the technique described by Goettel and Inglis [24]. Conidial suspension (0.1 mL) titrated to 3 × 10^6^ conidia mL^−1^ was spread-plated on 9-cm Petri dishes containing SDA medium. The percentage of germination was determined by counting the number of germinated conidia / 100 conidia in four separate areas per plate at ×200 magnification after incubation at 25 ± 2 °C for 20 h. Four replicate plates were used.

### 2.3. Quantification of Conidial Uptake by Fruit Flies from Treated Substrate

This experiment was carried out only on *C. cosyra *and intended to determine the number of conidia of *M. anisopliae* that a single fly was able to pick up following exposure to treated substrate. Inoculation of flies was done according to the technique described by Dimbi *et al*. [8]. One hundred (100) adult *C. cosyra* were exposed to fungus-treated velvet material covering the inner side of a cylindrical plastic tube (98 × 45 mm) that had the bottom removed and replaced by white nylon netting. Dry conidia (0.3 g) were spread evenly onto the velvet material before the flies were released into the tube. Flies were allowed to walk on the velvet material for three minutes after which they were transferred to Plexiglas cages (150 × 150 × 240 mm). Immediately after exposure, 10 flies were placed individually in a 2-mL plastic vial in which one mL of water containing 0.05% Triton X-100 was added. The tubes were then vortexed for 5 min to dislodge conidia from the insect’s body. The number of conidia was estimated using a hemocytometer. The process was repeated at 2-h intervals up to 8 h.

### 2.4. Transfer of Inoculum

Four groups of 20 two-week-old male *C. capitata*, *C. cosyra* and *C. fasciventris* were exposed to dry conidia of *M. anisopliae* using the technique described above. Twenty-four hours later, the treated male flies were mixed with equal numbers of 7-day-old fungus-free and unmated females and maintained together in Plexiglas cages (150 × 150 × 240 mm) for 24 h to allow for mating. A group of 20 fungus-free males and females were also held together in cages for 24 h and used as controls. Twenty-four hrs after exposure for mating, the flies were separated by sex and held in separate cages for 10 days at ambient temperatures (23–28 °C). Mortalities in both sexes were recorded. The experiment was replicated four times. In another set of experiment, four groups of two-week-old female *C. capitata*, *C. cosyra* and *C. fasciventris* were contaminated with fungal conidia as described above and then mixed with equal numbers of 7-day-old fungus-free and unmated males after 24 h. The experimental procedure was similar to that described previously. Control flies were also included. The experiment was also replicated four times.

### 2.5. Transfer of Inoculum through a Chain of Individuals

To evaluate the ability of fungus-infected flies to transfer fungal conidia to other healthy flies, a single female fly was exposed to dry conidia of *M. anisopliae* (~1.0 × 10^6^ conidia mL^−1^), as measured using a hemocytometer. The latter—also known as “donor”—was placed in a cage (150 × 150 × 240 mm) with three fungus-free males that served as “recipient” flies. Flies were allowed to mate and were separated thereafter and transferred individually to small cages (100 × 100 × 120 mm). Three additional fungus-free male flies were introduced every day for five consecutive days into a clean cage containing the “donor” female flies. Flies were transferred individually to clean cages each time the pairs were separated and later maintained at ambient conditions (23–28 °C). Mortality was recorded daily for 14 days. In the control treatments, flies were not treated with fungal conidia but the experimental procedure remained the same. A similar experiment was carried out with a male fly as the “donor” fly and female flies as the “recipient” flies following the procedure described above. The experiment was repeated four times.

### 2.6. Effect of Fungal Infection by M. anisopliae on Reproduction Potential

Fifteen females of each fruit fly species were exposed to conidia of *M. anisopliae *(~1.0 × 10^6^ conidia mL^−1^) and placed in Plexiglas cages (150 × 150 × 240 mm). An equal number of fungus-free male flies of the same species was added to each cage and held together for a period of 5–6 days when all treated females had died. The flies were fed as previously described. Ripe mangoes (approx. 140 mm long and 100 mm diam.) were used as substrate for oviposition. Each mango was cut into two halves; the pulp and seed were removed to give two hollow domes of the mango peel. Each sphere was placed in a 90-mm Petri dish lined with a damp black cloth (80 mm diam.) and later introduced into the cages. Eggs laid into the mango domes generally remained attached to the inner surface of the mango peels with a few dropping onto the damp cloth. The eggs were collected and counted daily under a dissection microscope. Female mortality was also recorded daily. Four replicates of 15 flies per cage were used in the experiment. To evaluate the effect of fungal infection on fertility, 20 eggs per treatment and per replicate were chosen at random each day and transferred to a 90-mm Petri dish lined with damp black cloth. Plates were incubated at room temperature (23–28 °C) and the number of eggs that hatched was recorded daily.

### 2.7. Data Analysis

In all tests, fly mortality due to fungal infection was adjusted for natural mortality in the controls using Abbott’s formula [25]. Mortality and fertility data were subjected to angular transformation before analysis. Daily egg production data were transformed to natural logarithms before analyses. Data were analyzed using the ANOVA procedure of SAS [26]. The number of eggs from treated and untreated females was compared using unpaired *t*-tests [27]. An alpha level of 0.05 was used for this comparison. Lethal time to 50% mortality (LT_50_) was estimated using logistic regression and analyses were carried out using the GENMOD procedure at 95% level of significance.

## 3. Results and Discussion

### 3.1. Retention of Conidia

The number of conidia picked up by a single *C. cosyra *fly immediately after removal from the contaminated substrate was 1.0 × 10^6^ conidia; but this number dropped from 7.2 × 10^5^ to 4.1 × 10^5^ conidia / fly, at 2 and 8 h, respectively (Table 1). The fungus-treated flies were observed to groom themselves more often than untreated ones. However, despite the grooming, flies were still able to retain as much as 4.3–4.1 × 10^5^ conidia per fly, 6 and 8 h after inoculation (Table 1), respectively. The fact that flies were able to retain some conidia after grooming has important implications on the horizontal transmission of inoculum to other members of the population.

**Table 1:** Number of conidia recovered from single *Ceratitis cosyra *flies after exposure to *Metarhizium anisopliae* and maintained for 2–8 h.

Time after treatment (h)	Mean number of conidia per fly
0	1.1 × 10^6^
2	7.2 × 10^5^
4	6.1 × 10^5^
6	4.3 × 10^5^
8	4.1 × 10^5^

### 3.2. Transfer of Inoculum

**Figure 1:**
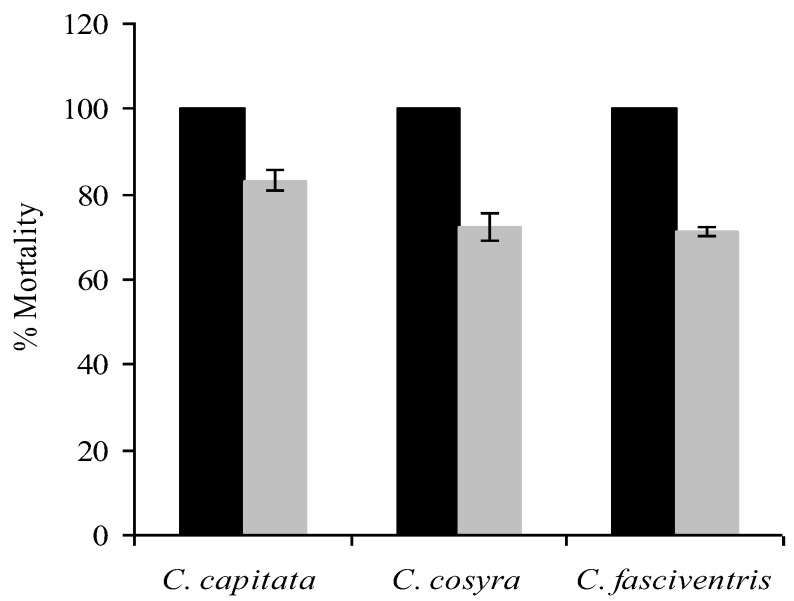
Horizontal transmission of *Metarhizium anisopliae* from treated male “donors” to free-fungus female “recipient” flies. Mortality observed after 10 days post-exposure. Bars denote means ± one standard error (*p =* 0.05).

**Figure 2:**
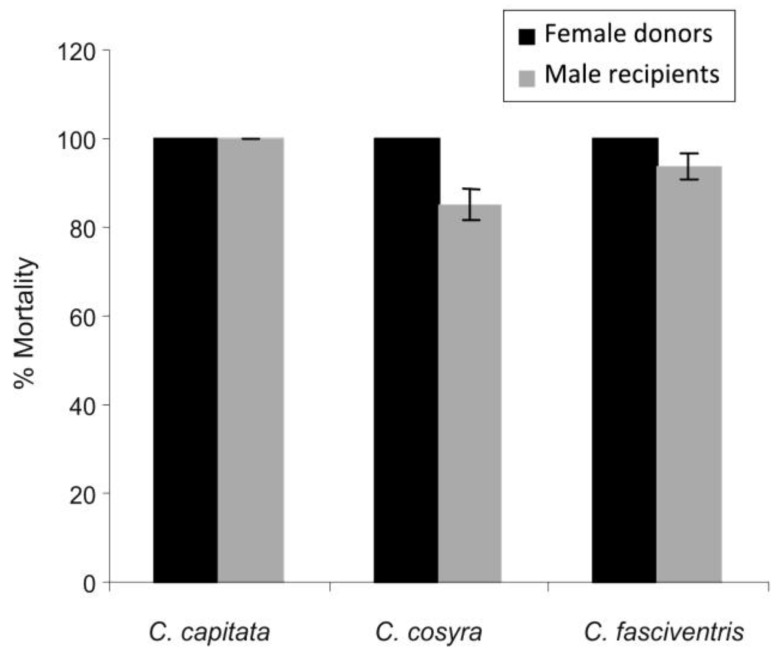
Horizontal transmission of *Metarhizium anisopliae* from treated female “donors” to fungus-free male “recipient” flies. Mortality observed after 10 days post-exposure. Bars denote means ± one standard error (*p =* 0.05).

In the viability test, 95% of conidia germinated after 20 h. Mortality in the controls did not exceed 10% in the three fruit fly species. The number of conidia picked up by a single “donor” fly varied between 3.8 × 10^5^ and 1.0 × 10^6^ while the “recipient” fly picked up between 1.0 × 10^5^ and 2.5 × 10^5^ conidia in the three fly species. Male and female *C. capitata*, *C. cosyra *and *C. fasciventris* exposed directly to conidia of *M. anisopliae* (“donors”) became infected and all died of fungal infection within 5–6 days post-exposure (Figure 1). They were able to transmit infection to fungus-free female “recipients” before death, resulting in a mortality of 83, 72 and 71% in *C. capitata*, *C. cosyra *and *C. fasciventris*, respectively, 10 days post-infection (Figure 1). Similarly, fungus-infected female fly “donors” transmitted infection to male “recipients”, resulting in mortalities of 100, 85 and 93% in *C. capitata*, *C. cosyra *and *C. fasciventris*, respectively, 10 days post-inoculation (Figure 2). In all three fruit fly species, the lethal time to 50% mortality (LT_50_) values ranged between 2.6 to 2.9 days in male “donor” flies and between 2.8 to 3.4 days in female “donor” flies; while it varied between 8.1 and 10.5 days in female “recipient” flies and between 5.6 and 7.9 days in male “recipient” flies of the three fly species (Table 2). Horizontal transmission of fungal infection from infected insects to healthy ones through mating or physical contact has been reported in dipterans including tsetse flies [11,12,17], mosquitoes [14], fruit flies [15,16] and root fly [13]. The ability to transmit infection from infected to uninfected host insects is one of the attributes of EPF and may, therefore, contribute to their success as biocontrol agents.

**Table 2:** Lethal time to 50% mortality (LT_50_) in days of “donor” and “recipient” flies after treatment of “donor” flies with *Metarhizium anisopliae*.

	LT_50_ day (X ± SE)		
	*C. capitata*	*C. cosyra*	*C. fasciventris*
Male “donors”	2.6 ± 0.1	2.9 ± 0.2	2.9 ± 0.1
Female “donor”	2.8 ± 0.1	3.4 ± 0.3	3.1 ± 0.1
Female “recipient”	8.1 ± 0.4	10.4 ± 0.4	10.5 ± 0.4
Male “recipient”	5.6 ± 0.5	7.9 ± 0.3	7.7 ± 0.2

### 3.3. Transfer of Inoculum through a Chain of Individuals

In all the three species of fruit flies, fungus-treated fly “donors” died within 3–6 days post-inoculation while “recipient” flies died within 5–14 days post-inoculation. Both fungus-treated male and female flies were able to pass on fatal doses of inoculum to at least three mating lines of flies of the opposite sex before they died. Similar results were reported by Meadow *et al.* [13] on cabbage root flies, *Delia radicum *L., with *Beauveria bassiana *(Bals.) Vuill. In that study, adult flies were able to receive and transmit fatal doses of inoculum from and to at least six flies in a chain. The fact that flies can mate more than once will obviously increase the chances of an infected fly transmitting an infection to several mates before it dies. Other mating behaviors of the flies could also contribute to enhance the transmission of the infection. For instance, female flies were observed to solicit courtships from a number of males before accepting one as a mate. After being mounted, the females would drop from the wall of the cages and aggressively shake off the unwanted male. Calling male flies in all the three species were also observed to make several attempts towards homosexual mating. Prokopy and Hendrichs [28] reported similar observations in courtship behavior of *C. capitata*. Such behavior has also been observed in calling males of the Queensland fruit fly, *Bactrocera tryoni *(Froggatt), where they mounted each other independent of sex and had many prolonged attempts at homosexual copulation [29]. This behavior observed in the African fruit flies in our study will evidently increase fly-to-fly transmission of the fungus. Additionally, in the field, male-to-male transmission could be enhanced when male flies aggregate during leks, a common phenomenon observed in the Mediterranean fruit fly [30,31].

### 3.4. Effect of Fungal Infection on Reproduction Potential

**Figure 3:**
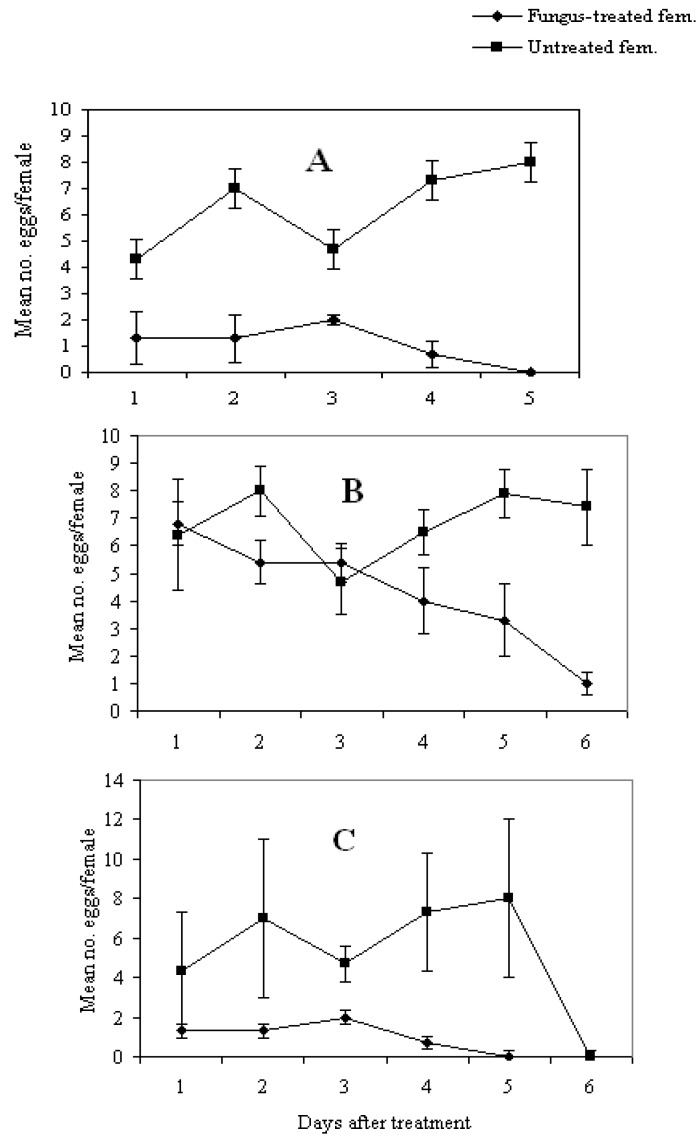
(**A**–**C**) Mean (X ± SE) number of eggs per female treated with *Metarhizium anisopliae*: A = *Ceratitis capitata*, B = *C. cosyra* and C = *C. fasciventris*.

Infection by *M. anisopliae* significantly affected egg laying in all the three species of flies tested. More eggs were laid by control females than by fungus-treated female flies (F = 9.83; df = 1.22; *p *= 0.004) in *C. capitata*, (F = 4.48; df = 1.27; *p *= 0.04) in *C. cosyra*, and (F = 6.77; df = 1.22; *p *= 0.01) in *C. fasciventris *(Figure 3). The percentage of reduction in fecundity was 82, 73 and 37% in *C. capitata*, *C. fasciventris *and *C. cosyra*, respectively*. *Similar results were reported by Castillo *et al.* [3] who obtained a 65% reduction in egg laying of *C. capitata* treated with *Isaria fumosorosea *(*=Paecilomyces fumosoroseus*) (Holm: Fries) Fries and 40–50% reduction when treated with *M. anisopliae* and *Aspergillus ochraceus *K. Wilh. Reduction in fecundity has also been reported in the onion maggot, *Delia antiqua* (Meigen), the carrot fly, *Psila rosae* Fabricius and the house fly, *Musca domestica *L. infected by *Entomophthora muscae *(Cohn) [18,19], and the African malaria vectors exposed to either *B. bassiana *or *M. anisopliae *[21,32,33]. Lower fecundity in fungus-infected females could be attributed to the invasive effects of fungal mycelia or the toxic effects of fungal metabolites or a combination of both [20]. However, according to Meadow *et al.* [13], the female flies might have been weakened by the infection. The reduction in oviposition is one of the benefits of fungal pathogens infecting pest insects as it would result in further reduction of the potential host populations and offset the low speed of kill of entomopathogenic fungi.

No significant difference in the hatchability of eggs was observed between the eggs from fungus-treated flies and untreated controls. Similar results were reported by Eilenberg [18] with *P. rosae *infected with *E. muscae.* However, Castillo *et al.* [20] reported reduced fertility of eggs of *C. capitata* with an isolate of *M. anisopliae*. 

## 4. Conclusions

This study has demonstrated that horizontal transmission of conidia between flies does occur during mating, which is fundamental to the autodissemination strategy being proposed for the management of African fruit flies. Field studies are now required to validate this concept in fruit flies, which is already documented for the tsetse fly [34].
